# Posterior Tibial Slope and a New Morphometric Method With Multiplanar Reconstruction Technique in a Turkish Sample

**DOI:** 10.7759/cureus.15472

**Published:** 2021-06-06

**Authors:** Ismail Eralp Kacmaz, Ali Er, Can Doruk Basa, Vadym Zhamilov, Mustafa Bozdag, Oguzhan Ekizoglu

**Affiliations:** 1 Orthopaedics, Tepecik Training and Research Hospital, Izmir, TUR; 2 Radiology, Tepecik Training and Research Hospital, Izmir, TUR; 3 Forensic Medicine, Tepecik Training and Research Hospital, Izmir, TUR

**Keywords:** tibia, slope, technique, arthroplasty, tomography, morphometry, turkish

## Abstract

Aim: The posterior tibial slope (PTS) is important in planning many orthopedic procedures. The aim of the study is to outline a PTS measurement method using multiplanar reconstruction (MPR) in knee computed tomography (CT) images.

Methods: MPR reconstruction was performed on pre-captured CT angio images of 124 patients. A standard tibial axis was created. Then, using reference points, the PTS was measured separately for the medial PTS (MPTS) and lateral PTS (LPTS). To identify an intra- and interobserver error, the technical error of measurement (TEM), relative TEM (rTEM), and coefficient of reliability (R) of the measurement were analyzed.

Results: The study enrolled 124 patients (88 males, 36 females) from 18 to 92 years old. The average MPTS 8.63 ± 2.7° and LPTS 7.77 ± 3.1° were significantly different (*p *< 0.05). However, there was no difference between the sexes (*p *= 0.52 for MPTS; *p *= 0.9 for LPTS). The R for intraobserver reliability was 0.942 for the MPTS and 0.943 for the LPTS, and that for interobserver reliability was 0.815 and 0.806, respectively.

Conclusions: PTS measurement from CT images appears advantageous as it eliminates measurement limitations due to tibial rotation and has high intra- and interobserver consistency.

## Introduction

The posterior tibial slope (PTS) is the angle between the lateral tibial plateau and longitudinal anatomical axis of the tibia in the sagittal plane [1]. The PTS differs among individuals and these interindividual differences affect the outcomes of orthopedic treatment. The clinical conditions most often affected by a high PTS are anterior cruciate ligament (ACL) injuries and those requiring prostheses. A high PTS increases the likelihood of ACL injury [2-4]. The PTS differs among populations, and these differences are important when designing total knee arthroplasty (TKA) implants [5,6].

PTS affects component positioning in TKA and maintenance of knee biomechanical balance in high tibial osteotomy (HTO) [7-11]. Incorrect component positioning after arthroplasty can lead to early loosening [8-12]. Instead of using a standard reference value, i.e., a slope of 7°, individual slope measurements that evaluate the medial and lateral plateaus separately cause fewer complications in HTO and unicondylar knee arthroplasty [8,13,14].

The method most commonly used for measuring PTS involves measuring the angle between the lateral tibial plateau and tibial longitudinal anatomical axis using reference points on lateral knee radiographs [14]. Recent improvements in cross-sectional imaging have led to new measurement techniques [15]. Magnetic resonance imaging (MRI) and computed tomography (CT) are now more widely used for morphometric analysis [16]. Imaging data can be stored in hospital databases for use in future investigations. Many researchers have attempted to devise new morphometric techniques based on the analysis of cross-sectional images of different parts of the skeleton [17]. Furthermore, morphometric measurements based on cross-sectional images can be standardized because anatomical features are easy to locate and identify on these images, especially when using CT with multiplanar reconstruction (MPR) or three-dimensional (3D) reconstruction [18-22].

This study investigated whether determining the angle between the lateral tibial plateau and longitudinal anatomical axis of the tibia based on CT images with MPR is applicable for assessing the PTS.

## Materials and methods

Computed tomography angiography (CTA) images of 165 patients who underwent multidetector lower extremity CTA in our institution from January 2015 to January 2019 were reviewed retrospectively to determine the PTS. Patients were referred for lower extremity CTA because of peripheral vascular pathology. We excluded all patients with a history of surgery or fracture, and those with any kind of implant around the knee (n = 12), radiographic signs of osteoarthritis (defined as ≥ grade III in the Kellgren-Lawrence classification system) (n = 23), or poor image quality, mainly due to technical problems or acute trauma around the knee (which can affect image interpretation and measurements; n = 6). Ultimately, the study enrolled 124 patients.

CTA was performed using a 128-slice CT scanner (SOMATOM Definition Edge, Siemens Healthcare, Erlangen, Germany). Scan parameters were 100 kV, 208 mA, section thickness 1 mm, and reconstruction interval 0.6 mm.

All data were transferred from the archive to a workstation (Aquarius workstation, TeraRecon, San Mateo, CA, USA) via internal network connections, providing MPR images. All measurements were performed by one orthopedic surgeon with 10 years of experience and one radiologist with 10 years of experience in musculoskeletal radiology. The measurement parameters used to evaluate PTS are described below.

**Figure 1:**
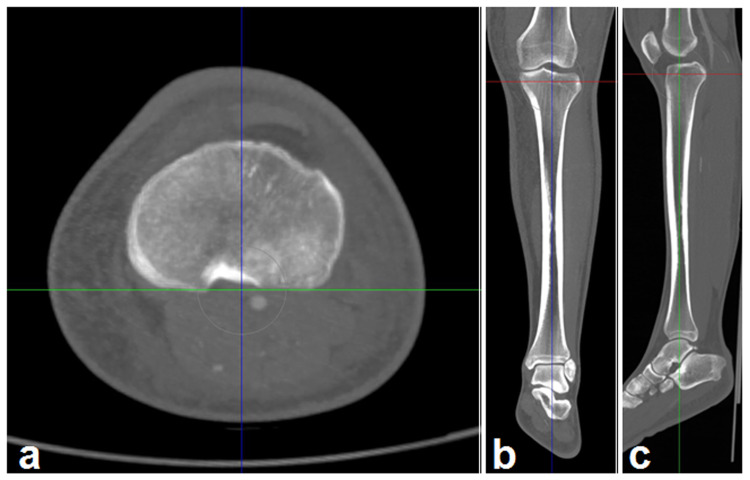
Determining the longitudinal axis of the tibia on axial (a), coronal (b), and sagittal (c) images

First, coronal and sagittal MPR images (slice thickness, 1 mm) were created from axial source images. Then, the tibia was made parallel to a reference line along the posterior margin of the tibial plateaus in the axial image (Figure 1a) to correct for tibial rotation. The correct axis was formed in the sagittal and coronal images created as a result of MPR. Then, the line passing through the middle section along the longitudinal long axis of the tibia was determined on axial, coronal, and sagittal MPR images (Figures 1a-1c). Thus, a standard axis was achieved in all three planes.

**Figure 2:**
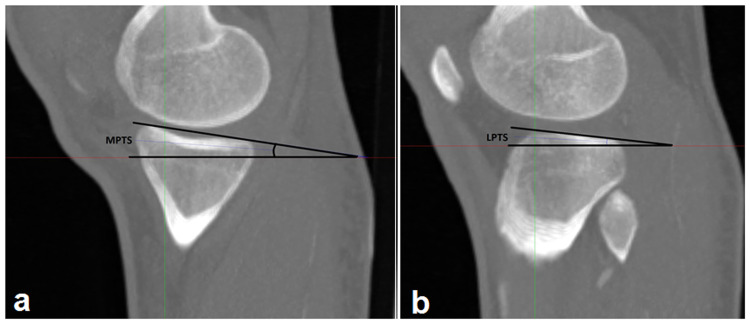
Measurement of medial posterior tibial slope (a) and lateral posterior tibial slope (b)

Then, by evaluating the sagittal images, the line connecting the highest points of the anterior and posterior tibia was determined in the section where the deepest point of the medial tibial plateau was seen. The medial PTS (MPTS) was determined by measuring the angle between this line and the line perpendicular to the tibial longitudinal axis ( Figure 2a). Similarly, the lateral PTS (LPTS) was measured on sagittal images with minimal lateral tibial plateau convexity (Figure 2b).

SPSS 22.0 (IBM, Armonk, NY, USA) was used to analyze the variables. A paired-samples t-test was performed to assess differences between the MPTS and LPTS. An independent-samples t-test was used to compare the MPTS and LPTS between groups based on sex. All differences were considered significant when p < 0.05. To assess intra- and interobserver error, two observers (orthopedic surgeon and radiologist) re-evaluated the MPTS and LPTS of all patients on CT images during two sessions in two weeks. The intra- (orthopedic surgeon) and interobserver (radiologist) errors were calculated using the technical error of measurement (TEM), relative TEM (rTEM), and coefficient of reliability (R) of the measurement, as suggested by Ulijaszek and Kerr [23].

The protocol for collecting the CTA images of human subjects was approved by the hospital ethics committee, and the study was conducted in accordance with the standards of the Declaration of Helsinki (Finland).

## Results

The study enrolled 124 patients aged 18 to 92 years, including 88 males (mean age 59.9 ± 13.2 years) and 36 females (mean age 63.5 ± 15.1 years). The average MPTS 8.63 ± 2.7° (range 1.3-14.9°) and LPTS 7.77 ± 3.1° (range 1-14.4°) were measured (Table 1). The difference between MPTS and LPTS was significant (p < 0.05); however, there was no difference between the sexes in either measurement (p = 0.52 for MPTS; p = 0.9 for LPTS).

**Table 1:** Distribution of average MPTS and LPTS by sex. MPTS: medial posterior tibial slope, LPTS: lateral posterior tibial slope, y: years

	Mean age (y)	Average MPTS	Average LPTS
Female (n:36)	63.5 ± 15.1	8.26 ± 2.9˚	8.21 ± 3˚
Male (n:88)	59.9 ± 13.2	8.78 ± 2.7˚	7.59 ± 3.1˚
Total (n:124)	60.9 ± 13.8	8.63 ± 2.7˚	7.77 ± 3.1˚

The intra- and interobserver error rates were also calculated for MPTS and LPTS using TEM, rTEM, and R. The intraobserver reliability was R = 0.942 for the MPTS and R = 0.943 for the LPTS. For the interobserver reliability, the error rates were R = 0.815 for the MPTS and R = 0.806 for the LPTS. Thus, the intra- and interobserver results were consistent throughout the study (Table 2).

**Table 2:** Estimation of intra- and inter-observer error using TEM, rTEM and R. TEM, technical error of measurement; rTEM, relative TEM; R; coefficient reliability, MPTS, medial posterior tibial slope; LPTS, lateral posterior tibial slope

		MPTS	LPTS
Intraobserver	TEM	0.591	0.652
	rTEM	6.425	8.393
	R	0.942	0.943
Interobserver	TEM	1.113	1.160
	rTEM	11.438	15.279
	R	0.815	0.806

## Discussion

Lateral knee radiography is used most commonly for measuring the PTS because of its short duration, low experience requirement, low radiation dose compared to CT, and low cost compared to other methods [1-24]. Many longitudinal axes have been used on lateral knee radiographs for measuring PTS. The lack of a standardized measurement method makes it difficult to compare data and clinical outcomes [6,25]. Measurement error due to tibial rotation can be as high as 13° [14,22,26]. Numerous authors have claimed that both plateaus need to be evaluated separately based on the large differences between cadaver and radiological studies of the PTS [4,22,27]. Separate evaluation of the plateaus confers an important advantage in preoperative planning of allograft application after unicondylar knee arthroplasty or tumor resection. Failure to distinguish the lateral and medial plateaus on direct X-rays due to superimposition is a measurement disadvantage [2,22,24]. It is thought that the disadvantages of X-ray-dependent measurements can be eliminated by using CT or MRI. Cross-sectional radiological examinations enable 3D analysis and visualization of different sections in one plane as well as better evaluation of cross-sectional morphology with MPR techniques [16,18,20,28,29].

As in measurement methods based on lateral knee radiography, the current CT and MRI studies continue to use different measurement methods. In a 3D-CT study, Zhang et al. measured the MPTS and LPTS using three different methods. They examined points 5 and 15 cm distal to the tibial plateau in sagittal sections. Three different longitudinal axes passing through the middle of the tibia, running parallel to the anterior cortex and running parallel to the posterior cortex, were identified. The values obtained with each method for all three angles were significantly different from each other [19]. In a CT study, Han et al. identified four different axes [6]. They measured the angles between the mechanical axis (MA), tibial anatomical axis (TAA), anterior tibial cortex (ATC), and fibular shaft axis (FSA) and reported that PTS measurements using CT can be used safely in TKA cases and that measurements made from the lateral tibia plateau may be more useful than those from the medial. The results of both CT studies support the MPTS and LPTS measurement differences and the efficacy of the CT measurement method we present here.

MRI appears useful for evaluating the medial and lateral tibial plateaus [6]. It requires no ionizing radiation, and the additional soft tissue evaluation can help clinical decisions during measurement. Simon et al. segmented 3D tibia images created using MRI images with AMIRA software (v4.1.2, Visage Imaging, Carlsbad, CA, USA) and measured the PTS using reference points similar to those described by Hashemi et al. [1,3]. They used the tibial spine to determine the tibial plateau boundaries. However, the tibial spine differs from person to person. This causes difficulty in determining reference points. With the MRI measurement method used by Hudek et al., cranial and caudal rings drawn according to the anterior and posterior cortex boundaries were identified in the images, and the longitudinal axis was determined based on the rings. Although this was a good technique for determining the longitudinal axis, rotation cannot be excluded because the entire tibia cannot be seen [20]. Our measurement method using CTA eliminates rotation-related limitations as it allows imaging of the entire tibia. The long axis of the tibia is detected exactly by MPR with CTA images. Although this method cannot be used routinely in patients undergoing CTA tibial slope evaluation, it can be used to determine the PTS in patients who require a diagnostic knee CT examination.

Kuwano et al. used the lateral tibial slope as a reference in patients undergoing TKA. They made measurements using 3D-CT, arguing that this is more consistent than measurements made with radiography or MRI. Especially in knees with osteoarthritis, the correct anteroposterior image could not be obtained due to the lack of full extension, so CT measurement was preferable [5].

In their study comparing different imaging methods, Naendrup et al. stated that the main limitation was not being able to exclude the effects of rotation defects, whereas we found that it is possible to minimize the measurement errors caused by rotation by using all axes when applying MPR [30].

Revealing the anatomy in more detail may be important for reducing observer-dependent errors. As in previous CT and MRI studies, our low intra- and interobserver errors may support this. Similarly, the low observer errors support the reliability of this approach in terms of the applicability and reproducibility of the defined methodologies. However, as with any new method, our technique should be evaluated by independent researchers in terms of operator-dependent errors. Operator experience must be considered when standardizing MPR measurements.

Comparing the results of morphometric analyses performed using different imaging techniques can help to refine the methods. Given the unique advantages and disadvantages of the various imaging modalities, studies should aim to help researchers choose the most appropriate method. Since our study was retrospective, different imaging methods could not be applied to the same patient. In addition, except for prospective diagnostic purposes, it is unethical to expose a patient to additional X-ray radiation or CT scans. In 2020, Naendrup et al. evaluated the correlations among radiography, MRI, and CT measurements of PTS [30]. There was a high degree of variability among, and inaccuracy in, the three imaging methods [16]. We recommend computed tomographic detection of parts of the anatomy, given its high sensitivity for determining standard reference points. In clinical practice, it may not be possible to apply our measurement method routinely, but it will be a useful option in some cases, including for arthroplasty, orthopedic oncology (for cases requiring major reconstruction), and revision ACL surgery, as well as in cases in which the evaluation is hampered by poor-quality X-ray images.

The main limitation of the methodology presented here is the ethical issue posed by the high radiation dose, which could be a barrier to its routine use in clinical practice. The image quality and clinical utility of low-dose radiation CT are remarkable. In particular, in anatomical evaluations of bone tissue, the quality of images obtained with low-dose CT may be sufficient for determining relevant anatomical landmarks and axes. Nevertheless, there are no analyses comparing the results with high- and low-dose CT.

In this study, two different observers re-evaluated the data, and the high intra- and interobserver agreement observed supports the reproducibility of the methodology. However, the CT cross-section on which the observers made their measurements was not specified in advance. This limits repeatability since it is not infeasible to use the same cross-section for each measurement. The sections that the observers evaluated, and the effects of difference therein on the results, could not be tested.

In this study, a new method for measuring the PTS based on CT with MPR was introduced. However, as this was a retrospective study, the measurements could not be compared to X-ray or MRI images of the same patients. Different imaging modalities and methods for measuring the PTS yield different values, i.e., there are discrepancies among methods [30].

## Conclusions

Our study obtained valuable data regarding clinical measurement of the PTS. Our method involves CT assessment of the lower extremities and eliminates the negative effects of tibial rotation associated with X-rays. However, it has the disadvantage of increased radiation exposure. Measuring the PTS using CT requires operator experience due to the difficulty in defining landmarks. Nonetheless, retrospective CT with MPR may be clinically useful, minimizing artifacts and allowing the degree of tibial rotation to be determined.
